# Prevalence and severity of frailty amongst middle-aged and older adults conveyed to hospital by ambulance between 2010 and 2017 in Wales

**DOI:** 10.1093/ageing/afaf124

**Published:** 2025-05-19

**Authors:** Carole Fogg, Tracey England, Helen Daniels, Bronagh Walsh

**Affiliations:** Faculty of Environmental and Life Sciences, University of Southampton, University Road, Southampton, Hampshire. SO17 1BJ, UK; Faculty of Environmental and Life Sciences, University of Southampton, University Road, Southampton, Hampshire. SO17 1BJ, UK; Population Data Science and Health Data Research UK, Swansea University, Swansea, West Glamorgan, UK; Faculty of Environmental and Life Sciences, University of Southampton, University Road, Southampton, Hampshire. SO17 1BJ, UK

**Keywords:** frailty, prehospital care, conveyance, older people

## Abstract

**Background:**

Ambulance services are commonly used by older adults. The scope of services continues to adapt in response to more non–life-threatening calls, often due to the acute consequences of chronic illness. Frailty increases with increasing age, but it is not known how common or severe it is within patients conveyed to hospital by ambulance.

**Methods:**

Open cohort of people aged ≥50 living in Wales between 2010 and 2017. Routinely collected electronic data on ambulance attendances resulting in conveyance were linked to primary care data within the Secure Anonymised Information Linkage databank, and the electronic Frailty Index was calculated. The prevalence and severity of frailty according to patient and incident characteristics was described.

**Results:**

Of 1 264 094 individuals within the cohort, 23.8% were taken to hospital between 2010 and 2017, of which frailty was present in 84.3% of patients. There was an upward trend in the number of conveyances for patients with moderate and severe frailty across the years in all age groups. The distribution of frailty was similar across call categories, deprivation quintiles and out-of-hours incidents. Patients conveyed from residential homes had a higher level of frailty and comprised 8.7% of the total conveyances.

**Conclusions:**

The high prevalence of frailty within adults aged ≥50 with emergency conveyances suggests upskilling ambulance crews with frailty training to enhance their assessment and decision making may improve patient outcomes. The high proportion of conveyances from residential homes indicates scope for increasing integration of community services to provide more patient-centred care pathways.

## Key Points

- The majority of adults aged ≥50 conveyed to hospital by ambulance have mild, moderate or severe frailty.
- Between 2010 and 2017, the overall number of conveyances of people with moderate and severe frailty increased by 81%.
- Approximately one-third of conveyances for people aged ≥50 are related to lower acuity (‘Green’) calls.
- Enhancement of ambulance crew education and competencies to include frailty may improve patient care and pathways.
- Continued development and spread of appropriate community services is needed to support lower acuity patients with frailty.

## Introduction

Frailty is a long-term condition in which multiple body systems gradually lose their in-built reserves [1]. Frailty is more common with advancing age and is associated with increased needs for health and care services [2, 3]. Estimates of frailty prevalence in different settings range from 38.9% in adults aged ≥50 in primary care [2], between 14.9% and 79.6% in unplanned hospital admissions [4], and 40% of all people aged ≥65 presenting to emergency departments (range 26%–51% between European sites) [5]. Understanding the prevalence in prehospital care is essential to ensure that appropriate services can be planned for people living with frailty, so that they receive the right care at the right time, e.g. integrated urgent care responses for non–life-threatening emergencies or conveyance to hospital [6, 7].

Models of care for older people with frailty and urgent care needs continue to be developed, with referral criteria, operating hours and context of available community care key considerations in implementation [8]. However, literature describing use of frailty assessments and subsequent care pathways by paramedics is limited. A cross-sectional study in patients aged ≥50 attended by paramedics estimated frailty prevalence at 58.7% [Clinical Frailty Scale (CFS) ≥5], with a 25% lower conveyance rate for frail patients [9]. However, an evaluation of paramedic use of the CFS in Canada showed good reliability but only moderate accuracy [10]. The use of the CFS by paramedics is not routine in Wales although potential frailty is flagged by key syndromes such as falls, changes in continence or delirium [11]. Elsewhere, performing a CFS may be prompted by a patient with reduced mobility or a fall, a diagnosis of dementia or features of the social context, but the issue of identifying and accessing appropriate onward care remains a problem [12, 13]. Additionally, secondary or community care–based frailty assessments or units are often limited to older age groups, whereas frailty is already common in midlife and may be severe [14, 15].

Understanding the scale of urgent care needs for people living with frailty is important to quantify the level of service provision and the required workforce to provide specialist emergency care in both the community and secondary care setting. A validated screening tool for frailty, the electronic Frailty Index (eFI), can be generated from the patient’s primary care record by identifying the presence and number of specific deficits including long-term health conditions, symptoms/signs, disabilities, abnormal laboratory test results and social conditions [16]. Linking primary care data including the eFI with ambulance records provides a unique opportunity to understand the extent of frailty within this population. This study aimed to estimate frailty prevalence and severity for patients conveyed to hospital by ambulance in a large cohort, to describe changes over the cohort period and to describe key patient and incident characteristics relevant to guiding recommendations [17].

## Methods

### Design

Retrospective cohort of routinely collected electronic health records (EHRs).

### Setting

Wales (UK) where 76% of GP practices submit EHR data to the Secure Anonymised Information Linkage (SAIL) databank, representing 79% of the Welsh population [18, 19]. Patients in Wales are registered with a single general practice within the primary healthcare system.

### Participants

(i) aged ≥50 years; (ii) registered at a general practice contributing to the SAIL databank; (iii) registered at any time between 2010 and 2017 (i.e. including patients turning 50 and those entering SAIL practices from other areas).

### Data sources

Patient records within the SAIL databank are pseudonymised and attributed a unique patient identifier which is the same across data sources. Primary care EHRs meeting eligibility criteria were identified and linked to ambulance conveyance records using the patient identifier.

### Measures

Frailty was assessed using the eFI [16]. Presence of the 36 eFI deficits was determined from associated Read codes within the electronic General Practice record, and the total deficits were divided by 36 to calculate the eFI for each eligible patient on 1 January of each calendar year in the cohort. The eFI was divided into categories reflecting increasing levels of frailty as per the original validation study, i.e. fit (eFI 0 to <0.12), mildly frail (0.12 to <0.24), moderately frail (0.24 to <0.36) or severely frail (≥0.36) [16]. As this measure is calculated from entries on the primary care record, it may underestimate frailty in people who are less likely to visit their General Practitioner (GP).

Additional variables included age, sex, indices of multiple deprivation quintiles, living in residential care and a diagnosis of dementia. Age was categorised reflecting literature relating to older adults’ healthcare, i.e. 50–64, 65–74, 75–84 and ≥85 years.

The number of conveyances, i.e. emergency calls for which patients were attended by a clinical ambulance team and conveyed to hospital, was calculated for each patient for each calendar year. Characteristics of calls included the main reason for the call, out-of-hours (6 p.m. to 8 a.m. Monday to Thursday, and 6 p.m. Friday to 8 a.m. Monday), call acuity attributed by 111/999 call handlers following a clinical algorithm: Red (immediately life-threatening), Amber (life-threatening or serious but not immediately life-threatening) or Green (not immediately serious or life-threatening) [20].

### Analysis

The number of people with at least one conveyance by age group and frailty category was calculated for each calendar year of the cohort and displayed graphically, and proportions of patient and incident characteristics according to frailty category for all conveyances were described using Stata (v 15.2; College Station, TX).

**Figure 1:**
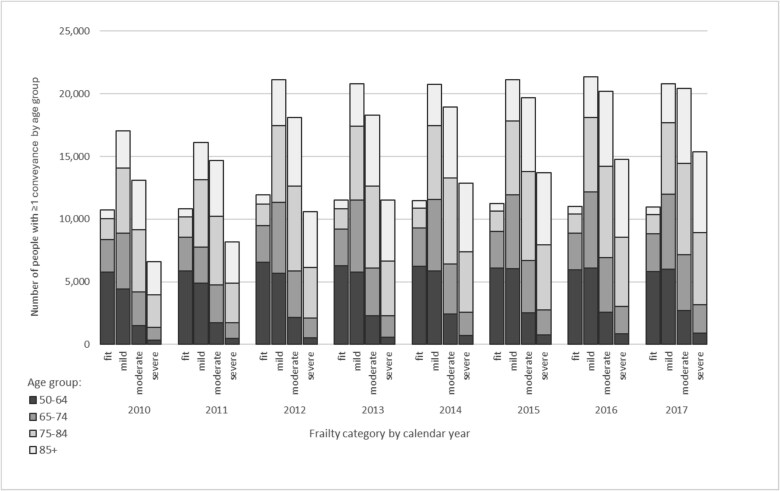
Number of people with at least one conveyance by age group and frailty category from 2010 to 2017.

## Results

The cohort increased from 895 551 patients in 2010 to 1 028 225 in 2017, with 1 264 094 patients contributing at least 1 year of data. The proportion aged ≥75 was 22% throughout. In 2010, 43% of the cohort were frail (30% mild, 10% moderate and 3% severe as per the eFI) and 56% were ‘fit’. The prevalence of frailty increased steadily, with 51% of patients being mildly, moderately or severely frail (33%, 13% and 5%, respectively) in 2017 and only 49% remaining fit.

There were 693 918 ambulance conveyances relating to 301 142 individual patients (23.8% of the cohort), 47% of whom were aged ≥75. Conveyances were recorded in 487 790 person-years, with 91% of years having either 1 or 2 conveyances and 7% with 3–5 conveyances. Over the 8-year period, the number of patients with conveyances who were not frail remained stable, as did the distribution of age groups within the fit category (Figure 1). Following an increase in conveyances in patients with mild frailty in 2012, these then also remained stable. However, the total number of conveyed patients with moderate or severe frailty increased by 81% between 2010 and 2017.

Overall, the prevalence of frailty in patients conveyed to hospital was 84.3% (Table 1). People with mild and moderate frailty comprised 61.6% of total conveyances. The proportion of conveyed patients with moderate and severe frailty increased as age increased, but 62.7% of patients aged 50–64 were already frail. Women comprised 54.4% of conveyances and tended to have higher levels of frailty than men. Patients in residential homes comprised 8.7% of conveyances, of whom 76.4% had moderate or severe frailty, with a similar pattern in patients with existing dementia diagnoses. There were ~1.5 times the number of conveyances of people living in the most deprived areas as compared to the least deprived; however, the prevalence and severity of frailty in conveyed patients was similar at all levels of deprivation.

**Table 1:** Prevalence and severity of frailty in patients conveyed to hospital according to patient and incident characteristics.

	Frailty category					
	Fit	Mild	Moderate	Severe	Total^a^	Total frail
**Total conveyances (*N*, %** ^**b**^ **)**	109 103, 15.7%	215 901, 31.1%	211 767, 30.5%	157 147, 22.6%	693 918	584 815, 84.3%
***Patient characteristics***						
**Age group**						
50–64	58 417, 37.3%	60 617, 38.7%	27 685, 17.7%	9830, 6.3%	156 549, *22.6%*	98 132, 62.7%
65–74	27 816, 18.0%	58 277, 37.8%	44 000, 28.5%	24 026, 15.6%	154 119, *22.2%*	126 303, 82.0%
75–84	15 832, 7.5%	61 127, 28.8%	76 191, 36.0%	58 775, 27.7%	211 925, *30.5%*	196 093, 92.5%
≥85	7038, 4.1%	35 880, 20.9%	63 891, 37.3%	64 516, 37.7%	171 325, *24.7%*	164 287, 95.9%
**Sex**						
Male	63 824, 20.2%	105 248, 33.3%	90 656, 28.7%	56 523, 17.9%	316 251, *45.6%*	252 427, 79.8%
Female	45 279, 12.0%	110 653, 29.3%	121 111, 32.1%	100 624, 26.6%	377 667, *54.4%*	332 388, 88.0%
**Living in a residential home**						
Yes	2447, 4.1%	11 790, 19.5%	21 226, 35.2%	24 866, 41.2%	60 329, *8.7%*	57 882, 95.9%
No	106 656, 16.8%	204 111, 32.3%	190 541, 30.1%	132 281, 20.9%	633 589, *91.3%*	526 933, 83.2%
**Dementia diagnosis**						
Yes	2818, 3.1%	18 186, 19.8%	32 685, 35.6%	38 194, 41.6%	91 883, *13.2%*	89 065, 96.9%
No	106 285, 17.7%	197 715, 32.8%	179 082, 29.7%	118 953, 19.8%	602 035, *86.8%*	495 750, 82.3%
**Index of multiple deprivation (quintile)**						
Quintile 1 (most deprived)	25 439, 15.4%	52 693, 31.8%	50 592, 30.5%	36 951, 22.3%	165 675, *23.9%*	140 236, 84.6%
Quintile 2	24 139, 16.1%	46 816, 31.2%	46 082, 30.7%	33 011, 22.0%	150 048, *21.6%*	125 909, 83.9%
Quintile 3	23 312, 15.5%	46 204, 30.7%	45 777, 30.5%	35 017, 23.3%	150 310, *21.7%*	126 998, 84.5%
Quintile 4	17 643, 15.4%	35 019, 30.6%	34 889, 30.5%	26 806, 23.4%	114 357, *16.5%*	96 714, 84.6%
Quintile 5 (least deprived)	18 570, 16.4%	35 169, 31.0%	34 427, 30.3%	25 362, 22.3%	113 528, *16.4%*	94 958, 83.6%
***Characteristics of incident***						
**10 most frequent reasons for the initial call** ^a^						
Health care professional^c^	9921, 28.6%	25 237, 34.9%	29 697, 39.6%	24 955, 42.7%	n/a–	–
Falls	7868, 22.7%	17 775, 24.6%	19 592, 26.1%	15 765, 27.0%	n/a	–
Sick person—specific diagnosis	3204, 9.2%	6649, 9.2%	6596, 8.8%	4752, 8.1%	n/a	–
Abdominal pain/problems	3052, 8.8%	4616, 6.4%	3462, 4.6%	2053, 3.5%	n/a	–
Stroke—CVA	1207, 3.5%	2288, 3.2%	2085, 2.8%	1353, 2.3%	n/a	–
Haemorrhage/lacerations	1114, 3.2%	2242, 3.1%	2110, 2.8%	1558, 2.7%	n/a	–
Traumatic injuries, specific	1109, 3.2%	1767, 2.4%	1567, 2.1%	1051, 1.8%	n/a	–
Breathing problems	1063, 3.1%	2393, 3.3%	2335, 3.1%	1694, 2.9%	n/a–	–
Overdose/poisoning (ingestion)	918, 2.7%	1094, 1.5%	n/a–	n/a–	n/a–	–
Unconscious/fainting (near)	903, 2.6%	1628, 2.3%	1409, 1.9%	915, 1.6%	n/a–	–
Diabetic problems	n/a–	n/a–	832, 1.1%	791, 1.4%	n/a–	–
**Call category**						
Red	41 043, 17.1%	78 327, 32.6%	71 934, 29.9%	49 267, 20.5%	240 571, *34.7%*	236 468, 98.3%
Amber	33 383, 15.7%	65 175, 30.6%	64 845, 30.5%	49 436, 23.2%	212 839, *30.7%*	179 456, 84.3%
Green	34 676, 14.4%	72 397, 30.1%	74 986, 31.2%	58 444, 24.3%	240 504, *34.7%*	205 828, 85.6%
**Out-of-hours**						
Yes	65 651, 16.1%	127 023, 31.2%	122 650, 30.1%	91 738, 22.5%	407 062, *58.7%*	341 511, 83.9%
No	43 452, 15.2%	88 878, 31.0%	89 117, 31.1%	65 409, 22.8%	286 856, *41.3%*	243 404, 84.9%

^a^Column percentages (italicised)

^b^Row percentages

^c^Indicates a health or care professional who has requested an ambulance following an initial assessment of the patient, including out-of-hours services

The most frequent reasons for the initial call to the emergency services were similar across frailty categories, although calls from a healthcare professional were more common in patients with moderate and severe frailty. A similar pattern of frailty prevalence and severity was observed in the three acuity levels of call category and in-hour versus out-of-hours calls. Lower category (‘Green’) calls comprised a third of conveyances; out-of-hours calls, nearly 60%.

## Discussion

Our results confirm the majority of patients aged ≥50 conveyed to hospital by ambulance are frail. One-fifth are severely frail, but a further 60% are living with mild or moderate frailty. Conveyances fall disproportionately amongst people aged ≥75, who comprise only 22% of the overall cohort but almost half of conveyances. Enhancing the skill set of paramedics to include acute frailty training and assessment may enable paramedics to make more informed decisions on whether to seek appropriate community-based alternative care to hospital or whether hospital conveyance is the best route. Alternatively, increased use of multidisciplinary emergency teams such as staff working in specialised falls and frailty emergency cars may offer an opportunity to manage patients safely at home [11, 21].

Front-door frailty services such as those based within the emergency department or a hospital-based same day emergency care normally cater for people above a certain age (e.g. ≥70) [22, 23]. However, the large numbers of people with mild or moderate frailty conveyed to hospital, including adults aged 50–74, present an opportunity for assessment and/or referral whilst in hospital, with increased integration with primary care or multidisciplinary community teams. Earlier initiation of proactive care planning for patients at risk of frailty progression at younger ages and/or earlier stages of frailty may reduce future emergency attendances or hospital conveyances, especially for frequent users of ambulance services, with resulting improvement in patient care and reduction of costs.

The increase in conveyances of people with moderate and severe frailty during the study suggests a shift in the type of population needing ambulance services, which may be driven by shortfalls in care [24], increasing complexity and severity of illness in a frailer, older population [25, 26]. Additionally, the large numbers of conveyances for people with non–life-threatening conditions, of which 60% were for people with mild or moderate frailty, may have been managed in the community if appropriate services were available [6, 27].

### Strengths and limitations

This is the first analysis to describe frailty in a large cohort of older adults conveyed to hospital by ambulance and demonstrates a significant need for prehospital urgent care. However, trends seen here may have preceded reconfigurations in community care and expansion of urgent care services for people living with frailty, so study of recent data which also includes referrals for patients not conveyed is recommended to understand the potential impact on care pathways. The eFI was indicated to overestimate frailty when compared to the CFS in one study [28], although it has demonstrated convergent validity in a larger population [29]. However, all frailty scores or indices should be used as a screening tool with subsequent detailed assessment, so the choice of a tool should be guided by what is feasible in the care setting.

### Future research

Evaluating the impact of frailty-specific interventions in the ambulance setting, such as education, use of assessments and extension of frailty management skills, is essential to provide an evidence base for effective care. Likewise, examining outcomes for patients who are not conveyed to hospital after receiving community-based care is key to quantify benefits and identify unforeseen risks in changes of service configurations. Lastly, although ambulance attendances to older people living in care homes are less likely to result in conveyances than community attendances [30], the significant numbers of conveyances from residential care found in our study merits exploration to understand factors influencing conveyance decisions, e.g. care coordination between professional groups and availability of end-of-life care.

## Conclusions

The high prevalence of frailty within adults aged ≥50 with emergency conveyances, particularly in low acuity calls, suggests upskilling ambulance crews with frailty training to enhance their assessment and decision making may improve patient outcomes. The high proportion of conveyances from residential homes indicates scope for increasing integration of community services to provide more patient-centred care pathways.
